# Transcriptomic Analysis Reveals LncRNAs Associated with Flowering of *Angelica sinensis* during Vernalization

**DOI:** 10.3390/cimb44050128

**Published:** 2022-04-26

**Authors:** Xiaoxia Liu, Mimi Luo, Mengfei Li, Jianhe Wei

**Affiliations:** 1 State Key Laboratory of Aridland Crop Science, College of Life Science and Technology, Gansu Agricultural University, Lanzhou 730070, China; 18893481962@163.com (X.L.); luomimi9521@163.com (M.L.); 2 Institute of Medicinal Plant Development, Chinese Academy of Medical Sciences & Peking Union Medical College, Beijing 100193, China

**Keywords:** *Angelica sinensis*, flowering, lncRNA, vernalization, transcriptomic analysis

## Abstract

*Angelica sinensis* is a “low-temperature and long-day” perennial plant that produces bioactive compounds such as phthalides, organic acids, and polysaccharides for various types of clinical agents, including those with cardio-cerebrovascular, hepatoprotective, and immunomodulatory effects. To date, the regulatory mechanism of flowering under the photoperiod has been revealed, while the regulatory network of flowering genes during vernalization, especially in the role of lncRNAs, has yet to be identified. Here, lncRNAs associated with flowering were identified based on the full-length transcriptomic analysis of *A. sinensis* at vernalization and freezing temperatures, and the coexpressed mRNAs of lncRNAs were validated by qRT-PCR. We obtained a total of 2327 lncRNAs after assessing the protein-coding potential of coexpressed mRNAs, with 607 lncRNAs aligned against the TAIR database of model plant *Arabidopsis*, 345 lncRNAs identified, and 272 lncRNAs characterized on the SwissProt database. Based on the biological functions of coexpressed mRNAs, the 272 lncRNAs were divided into six categories: (1) chromatin, DNA/RNA and protein modification; (2) flowering; (3) stress response; (4) metabolism; (5) bio-signaling; and (6) energy and transport. The differential expression levels of representatively coexpressed mRNAs were almost consistent with the flowering of *A. sinensis*. It can be concluded that the flowering of *A. sinensis* is positively or negatively regulated by lncRNAs, which provides new insights into the regulation mechanism of the flowering of *A. sinensis*.

## 1. Introduction

*Angelica sinensis* (Oliv.) Diels is a “low-temperature and long-day” perennial plant that is native to Gansu Province, Northwest China [1,2]. The roots have been used as a traditional Chinese medicine for over 2000 years [3,4]. Currently, the roots are also applied in cardio-cerebrovascular, hepatoprotective, and immunomodulatory clinical agents, largely relying on bioactive compounds such as phthalides, organic acids, and polysaccharides [5,6,7].

Recently, the planting area of *A. sinensis* has exceeded 40,000 ha to satisfy the increasing demand; however, a higher rate (>40%) of early bolting and flowering (EBF) in commercial cultivation increases the lignified rate of roots and decreases the yield accordingly [8,9]. In order to inhibit the EBF, effective measures that have been taken include selecting excellent germplasm resources with a lower rate of EBF [8], controlling the seedling size (0.4 to 0.6 cm) to delay the transition from vegetative to reproductive growth [10], storing seedlings below freezing temperatures (−3 to −10 °C) to avoid vernalization (0 to 10 °C) [2,11,12], and shading the plants with sunshade nets (40% to 60%) to avoid the long-day conditions during the adult stages [13].

Regarding the regulatory mechanism of flowering in *A. sinensis*, key genes and the regulatory network during the photoperiod have been identified and mapped based on transcriptomic analysis. Specifically, 13 genes associated with the photoperiod, vernalization, sucrose, and GA pathways were identified from plants at the vegetative stage compared with the EBF stage [14]; 38 genes associated with the photoperiod, carbohydrates, hormone signaling, and floral development were identified from different development stages [15]; and 40 genes associated with the photoperiod, sucrose, GA, and floral development were identified from the EBF compared with Un-EBF [16]. In summary, key genes such as *FLC*, *SOC1*, *FT*, *PHYA*, *AP1*, and *GA_2_OX_1_* were identified during the transition from the vegetative to the reproductive stage [14,15,16]. To date, physiological changes in the levels of carbohydrates, proteins, and hormones during vernalization have been investigated [17,18], while the regulatory network of flowering has yet to be identified.

Generally, ncRNAs include two categories, housekeeping and regulatory ncRNAs, and the latter can be further divided into sRNAs (i.e., miRNA, siRNA, and piRNA) and lncRNAs (>200 nucleotides) [19]. Both miRNAs and lncRNAs can influence plant developmental processes and stress responses [20], with the former being negative regulators functioning as specificity determinants, or guides, within complexes that promote the degradation of mRNA targets, and the latter acting either as precursors of miRNAs or endogenous target mimics (TMs), which mimic the real targets of miRNAs, thus rendering the corresponding miRNAs ineffective [21]. For example, previous studies on resistance against leaf rust in wheat found that 50 miRNAs and 1178 lncRNAs were identified and 49 lncRNAs were found to be the targets for miRNAs, with 1 lncRNA acting as a precursor of 2 miRNAs, and 3 lncRNAs acting as TMs [22].

Extensive investigations have demonstrated that lncRNAs regulate their downstream targets’ expression through the changing of epigenetic modification at the level of transcription and post-transcription by interacting with DNA, RNA, and proteins; thus, they are involved in various biological processes [23,24,25]. In this study, lncRNAs associated with flowering were identified based on the transcriptomic analysis of *A. sinensis* seedlings treated at vernalization and freezing temperatures (avoiding vernalization). We found that 272 lncRNAs directly or indirectly participate in regulating the flowering of *A. sinensis* and stress responses.

## 2. Materials and Methods

### 2.1. Plant Material

The seedlings (root–shoulder diameter 0.4–0.5 cm; Figure S1) of *Angelica sinensis* (cultivar Mingui 1) were stored at 0 (vernalization temperature) and −3 °C (freezing temperature), respectively. After storage at 0 °C for 14 (T1) and 60 days (T2), as well as at −3 °C for 125 days (T3), the shoot apical meristem (SAM) was cut from the root shoulder of the seedlings for transcriptomic analysis and qRT-PCR validation. Three biological replicates were performed for each treatment of T1, T2, and T3. Herein, the treatment of T1, T2, and T3 represents uncompleted, completed, and avoided vernalization, respectively, based on the EBF rate (Figure S2) when the stored seedlings were cultivated and grown in a long-day condition.

### 2.2. Full-Length Isoform Sequencing and Analysis

Total RNA of the SAM samples was extracted using Trizol reagent (Omega Bio-Tek, Norcross, GA, USA). The integrity of the RNA was determined using an Agilent 2100 Bioanalyzer (Agilent Technol., California, CA, USA) and agarose gel electrophoresis, and the purity and concentration of the RNA were determined using a microspectrophotometer (NanoDrop Technol., Wilmington, DE, USA). The high-quality RNAs were sequenced on a Pacific Biosciences Sequel platform (Gene Denovo Biotechnology Co., Ltd., Guangzhou, China). Raw reads of cDNA library were analyzed using a SMRT Link (V8.0.0) [26]. Briefly, high-quality CCS were extracted from the subreads BAM file; the integrity of transcripts (full-length sequences) was judged based on whether CCS reads contained primers (5′ and 3’) and polyAs; then, FLNC reads were generated by removing primers, barcodes, and polyAs; finally, FLNC reads were assembled to obtain the entire isoform [27].

### 2.3. Analysis of Long Noncoding RNAs (lncRNAs)

Isoforms that were not annotated against the four databases—NR, Swiss-Prot, Kyoto KEGG, and KOG—were used for the analysis of lncRNAs. The isoform that was assessed as a noncoding transcript by both CNCI and CPC software was finally confirmed as a lncRNA [28,29].

### 2.4. Characterization of LncRNAs

To date, the genome of *A. sinensis* has not been sequenced. Thus, the lncRNA analysis of *A. sinensis* was performed via a BLAST search with an E-value cut-off of ≤1 × 10^−5^ against the known lncRNAs from the TAIR database (https://www.arabidopsis.org accessed on 30 March 2022) [30]. The function of lncRNAs was annotated based on their coexpression mRNAs [31,32,33]. Herein, the biological functions of the coexpressed mRNAs were searched on the UniProt database (https://www.uniprot.org accessed on 30 March 2022).

### 2.5. qRT-PCR Validation

Based on the coding sequences (CDS) of coexpressed mRNA of lncRNA, 49 primer sequences of representatively coexpressed mRNAs (Table 1) were designed using the NCBI primer-blast tool. First-strand cDNA synthesis and qRT-PCR reaction were carried out using SuperRealPreMix Plus (FP205; Tiangen Biotech., Beijing, China) according to the manufacturer’s instructions; specifically, the cDNA was synthesized successively with one cycle (95 °C, 15 min) and 40 cycles (95 °C, 10 s; 60 °C, 20 s; and 72 °C, 30 s), and the qRT-PCR reaction was incubated successively at 95 °C for 15 s, 60 °C for 1 min and 95 °C for 1 s. The *Actin* (*ACT*) gene was used as a reference control gene with forward: TGGTATTGTGCTGGATTCTGGT and reverse: TGAGATCACCACCAGCAAGG (amplicon size 109 bp) [34]. Herein, the cycle threshold (Ct) values and standard curves of the ACT gene at different volumes (0.25, 0.5, 1.0, 1.5, 2.0, and 3.0 μL) was built to correct the gene expression level (Figure S3 and Figure S4), and the expression levels of the 49 candidate genes and their standard deviations for every variant were added to the Supplementary Materials (Table S1). The REL of coexpressed mRNAs was calculated using the 2^−^^△△^^Ct^ method [35] according to the following formula.

△Ct_Test gene_ = Ct_Test gene_ − Ct_Reference gene_

△Ct_Target gene_ = Ct_Target gene_ − Ct_Reference gene_

−△△Ct_(T2 vs. T1)_ = −(△Ct_T2_ − △Ct_T1_)

−△△Ct_(T3 vs. T1)_ = −(△Ct_T3_ − △Ct_T1_)

Relative expression level (REL) = 2^−^^△△^^Ct^.

### 2.6. Statistical Analysis

In order to obtain the precise estimation of PCR efficiency, each experiment for qRT-PCR validation was performed with three biological replicates, along with three technical replicates [36]. A t-test in SPSS 22.0 was performed for independent experiments, with *p* < 0.05 as the basis for statistical differences.

## 3. Results

### 3.1. LncRNAs Analysis

**Figure 1:**
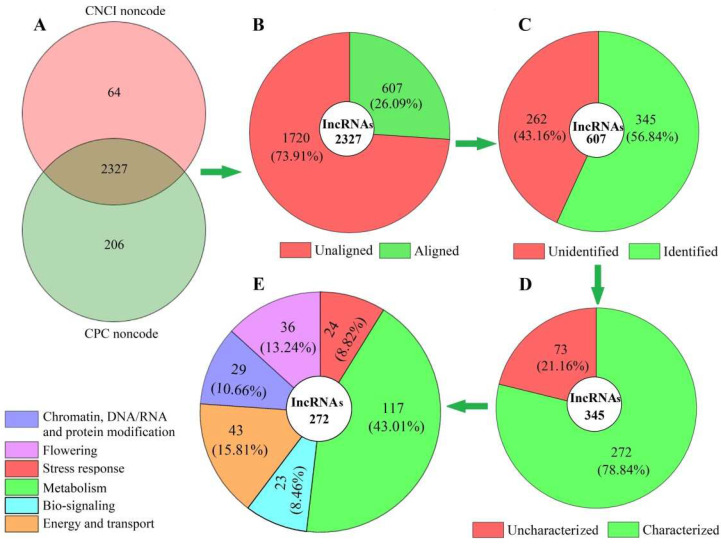
Distribution and classification of lncRNAs in *Angelica sinensis* during vernalization, based on the biological functions of coexpressed mRNAs. Abbreviations: CNCI, coding–noncoding index; CPC, coding potential calculator. Images (**A**), (**B**), (**C**), (**D**) and (**E**) represent total, aligned, identified, characterized and classified lncRNAs, respectively.

In total, 2327 lncRNAs were obtained after assessing the protein-coding potential of coexpressed mRNAs based on the two software programs, CNCI and CPC (Figure 1A), with 607 genes aligned against the known lncRNAs from the TAIR database of model plant *Arabidopsis* (Figure 1B), and 345 lncRNAs with coexpressed mRNAs of *A. sinensis* identified (Figure 1C) based on the SwissProt database. Based on the biological functions, the 272 characterized lncRNAs (Figure 1D) were divided into six categories: chromatin, DNA/RNA and protein modification (29); flowering (36); stress response (24); metabolism (117); biosignaling (23), and energy and transport (43) (Figure 1E). The base sequences of the 272 lncRNAs are shown in Table S2.

### 3.2. LncRNAs Linked with Chromatin, DNA/RNA and Protein Modification, as well as Expression Levels of Their Coexpressed mRNAs

**Figure 2:**
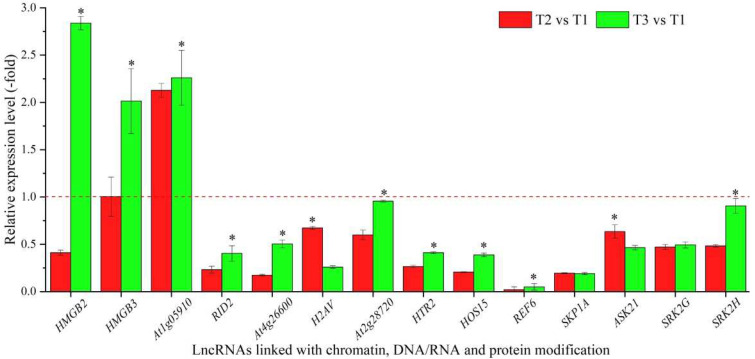
The expression levels of coexpressed mRNAs of lncRNAs linked with chromatin, DNA/RNA and protein modification in *A. sinensis* at T2 vs. T1 and T3 vs. T1, as determined by qRT-PCR. T1, T2, and T3 represent uncompleted, completed, and avoided vernalization, respectively. The “*” represents a significant difference at *p* < 0.05 level between T2 vs. T1 and T3 vs. T2 for the same gene.

Based on the biological functions of coexpressed mRNAs, 29 lncRNAs were linked with chromatin (*HMGB2* and *HMGB3*), DNA/RNA (*At1g05910, RID2* and *At4g26600*) and protein modification (*H2AV, At2g28720, HTR2, HOS15, REF6, SKP1A, ASK21, SRK2G, SRK2H, DET1, BOPAt4g295601, At1g45180, At3g50840, At2g36630, At3g47890, UBP7, DER2.1, GRP3, MDH9.13, At3g24715, At3g16560, At3g62260, ESMD1,* and *At1g27930*) (Table 2). The expression levels of 14 representative coexpressed mRNAs were confirmed by qRT-PCR, with 3 mRNAs (HMGB2, HMGB3 and At1g05910) showing down-regulation at T2 versus (vs.) T1, and 11 mRNAs showing lower levels at T2 vs. T1 than T3 vs. T1, with the exception of 2 mRNAs (H2AV and ASK21) (Figure 2).

### 3.3. LncRNAs Linked with Flowering and Expression Levels of Their Coexpressed mRNAs

**Figure 3:**
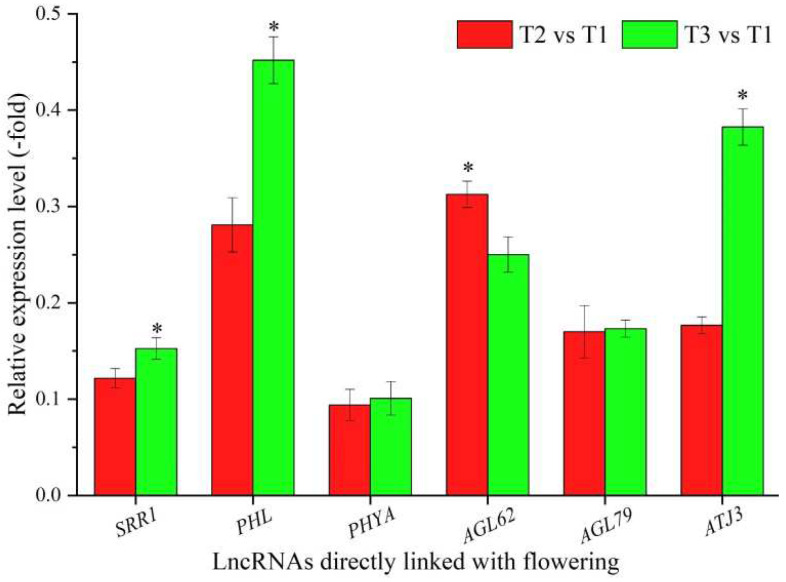
The expression levels of coexpressed mRNAs of lncRNAs directly linked with flowering in *A. sinensis* at T2 vs. T1 and T3 vs. T1, as determined by qRT-PCR. The “*” represents a significant difference at *p* < 0.05 level between T2 vs. T1 and T3 vs. T2 for the same gene.

In total, 36 lncRNAs were linked with flowering based on the biological functions of their coexpressed mRNAs, with 12 lncRNAs directly associated with flowering, namely *SRR1*, *PHL*, *PHYA*, *AGL62*, *AGL79*, *ATJ3*, *BBX29*, *CLE13*, *CLE44*, *MXC17.10*, *At1g06515*, and *BHLH30* (Table 3), and 24 lncRNAs indirectly associated with flowering, such as cell division, embryo development, and cell wall organization (Table S3). The expression levels of six representative coexpressed mRNAs (*SRR1*, *PHL*, *PHYA*, *AGL62*, *AGL79*, and *ATJ3*) were confirmed by qRT-PCR, with all six mRNAs showing down-regulation at T2 vs. T1 and T3 vs. T1, and five mRNAs showing lower levels at T2 vs. T1 than T3 vs. T1, with the exception of the gene *AGL62* (Figure 3).

### 3.4. LncRNAs Linked with Stress Response and Expression Levels of Their Coexpressed mRNAs

**Figure 4:**
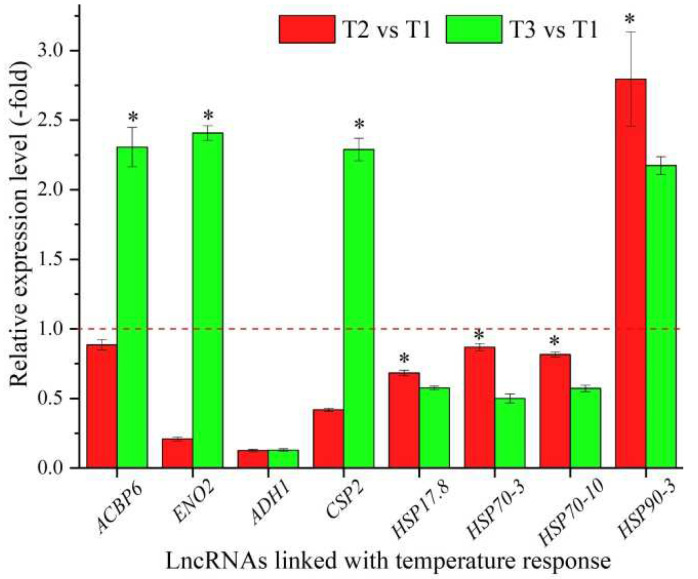
The expression levels of coexpressed mRNAs of lncRNAs linked with temperature response in *A. sinensis* at T2 vs. T1 and T3 vs. T1, as determined by qRT-PCR. The “*” represents a significant difference at *p* < 0.05 level between T2 vs. T1 and T3 vs. T2 for the same gene.

In total, 24 lncRNAs were linked with the stress response based on the biological functions of their coexpressed mRNAs, with 14 lncRNAs associated with the temperature response, namely *ACBP6*, *ENO2*, *ADH1*, *CSP2*, *RH20*, *RH52*, *RH53*, *RAB18*, *XERO1*, *MED14*, *HSP17.8*, *HSP70-3*, *HSP70-10*, and *HSP90-3* (Table 4), and 10 lncRNAs associated with other stresses responses, such as water, salt, and oxidative stress (Table S4). The expression levels of eight representative coexpressed mRNAs involved in the temperature response were confirmed by qRT-PCR, with four mRNAs (*ACBP6*, *ENO2*, *CSP2*, and *HSP90-3*) showing up-regulation at T3 vs. T1, and three mRNAs (*ACBP6*, *ENO2*, and *CSP2*) showing lower levels at T2 vs. T1 than T3 vs. T1 (Figure 4).

### 3.5. LncRNAs Linked with Metabolism and Expression Levels of Their Coexpressed mRNAs

**Figure 5:**
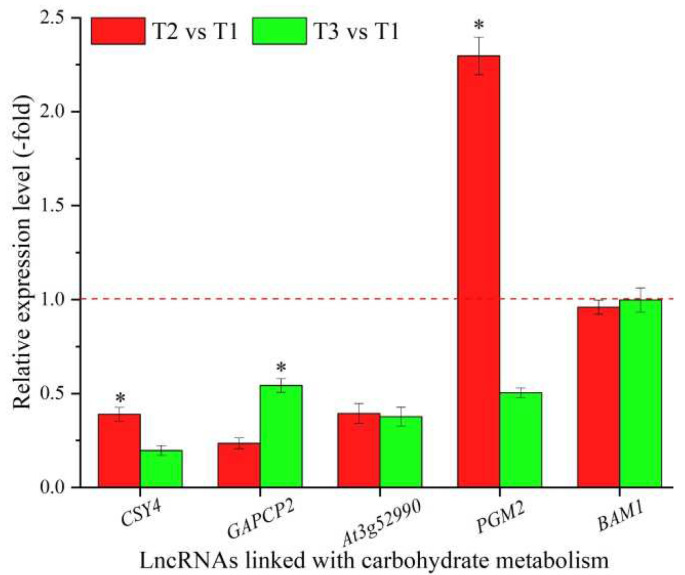
The expression levels of coexpressed mRNAs of lncRNAs linked with carbohydrate metabolism in *A. sinensis* at T2 vs. T1 and T3 vs. T1, as determined by qRT-PCR. The “*” represents a significant difference at *p* < 0.05 level between T2 vs. T1 and T3 vs. T2 for the same gene.

In total, 117 lncRNAs were linked with metabolism based on the biological functions of their coexpressed mRNAs, with 19 lncRNAs associated with carbohydrate metabolism, namely *CSY4*, *FBA3*, *GAPC1*, *GAPCP2*, *At3g52990*, *PGM2*, *USP*, *GLCNAC1PUT2*, *UXS2, XYLA*, *GALS1*, *OFUT31*, *At5g67460*, *RSW3*, *At1g59950*, *At5g25970*, *UGT76E7*, *At1g26850*, and *BAM1* (Table 5), and 98 lncRNAs associated with other types of metabolism, such as nucleotide, protein, and lipid metabolism (Table S5). The expression levels of five representative coexpressed mRNAs (*CSY4*, *GAPCP2*, *At3g52990*, *PGM2*, and *BAM1*) involved in carbohydrate metabolism were confirmed by qRT-PCR, with all five mRNAs showing down-regulation at T3 vs. T1, and three mRNAs showing higher levels at T2 vs. T1 than T3 vs. T1, with the exception of the two genes *GAPCP2* and *BAM1* (Figure 5).

### 3.6. LncRNAs Linked with Biosignaling and Expression Levels of Their Coexpressed mRNAs

**Figure 6:**
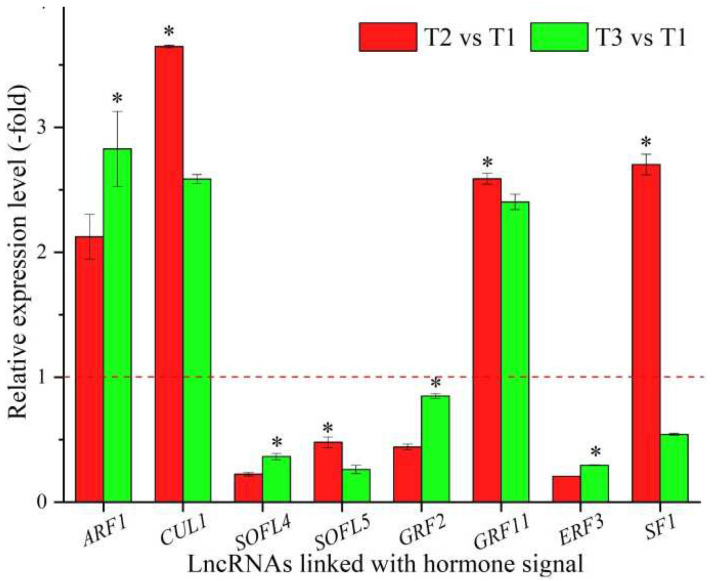
The expression levels of coexpressed mRNAs of lncRNAs linked with biosignaling in *A. sinensis* at T2 vs. T1 and T3 vs. T1, as determined by qRT-PCR. The “*” represents a significant difference at *p* < 0.05 level between T2 vs. T1 and T3 vs. T2 for the same gene.

In total, 23 lncRNAs were linked with metabolism based on the biological functions of their coexpressed mRNAs, with 13 lncRNAs associated with hormone signaling, namely *ARF1*, *CUL1*, *T4L20.330*, *SOFL4*, *SOFL5*, *GRF2*, *GRF11*, *ERF3*, *CIPK20*, *SF1*, *AGD9*, *TIFY4B*, and *At2g34810* (Table 6), and 10 lncRNAs associated with other types of signaling, such as protein kinase, phosphatidylinositol-mediated, and cell surface receptor signaling (Table S6). The expression levels of eight representative coexpressed mRNAs associated with hormone signaling were confirmed by qRT-PCR, with three mRNAs (*ARF1*, *CUL1*, and *GRF11*) showing up-regulation at T2 vs. T1 and T3 vs. T1, and four mRNAs (*ARF1*, *SOFL4*, *GRF2*, and *ERF3*) showing lower levels at T2 vs. T1 than T3 vs. T1 (Figure 6).

### 3.7. LncRNAs Linked with Energy and Transport

**Figure 7:**
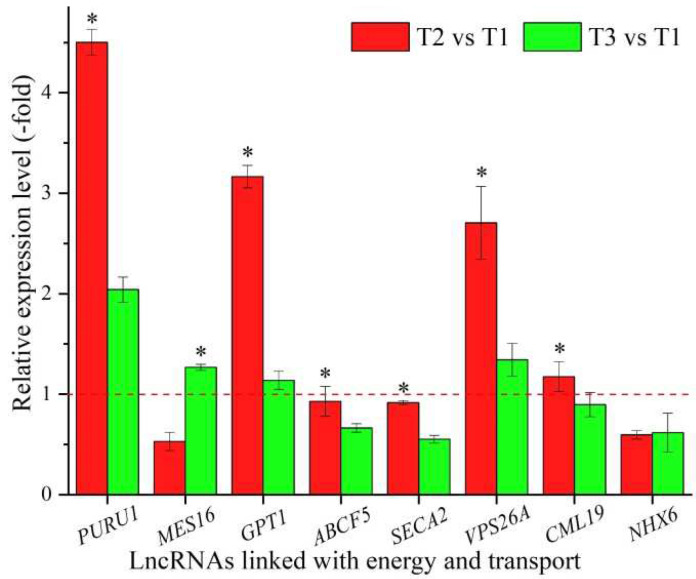
The expression levels of coexpressed mRNAs of lncRNAs linked with energy and transport in *A. sinensis* at T2 vs. T1 and T3 vs. T1, as determined by qRT-PCR. The “*” represents a significant difference at *p* < 0.05 level between T2 vs. T1 and T3 vs. T2 for the same gene.

In all, 43 lncRNAs were found to be linked with energy and transport based on the biological functions of their coexpressed mRNAs, with 5 lncRNAs associated with energy, such as *PURU1*, *ndhB1*, and *NDB1*, and 38 lncRNAs associated with transport, such as *At5g11230*, *GPT1* and *SMXL5* (Table 7). The expression levels of eight representative coexpressed mRNAs (*PURU1*, *MES16*, *GPT1*, *ABCF5*, *SECA2*, *VPS26A*, *CML19*, and *NHX6*) were confirmed by qRT-PCR, with three mRNAs (*PURU*, *GPT1*, and *VPS26A*) showing up-regulation at T2 vs. T1 and T3 vs. T1, and six mRNAs (*PURU1*, *GPT1*, *ABCF5*, *SECA2*, *VPS26A*, and *CML19*) showing higher levels at T2 vs. T1 than T3 vs. T1, with the exception of the two genes *MES16* and *NHX6* (Figure 7).

## 4. Discussion

Vernalization is a process considered to be an epigenetic switch whereby flowering is promoted by prolonged exposure to cold (0 to 10 °C); meanwhile, it can be lost at high temperatures or avoided below freezing temperatures [37,38]. Epigenetic regulation involves diverse molecular mechanisms including chromatin remodeling, DNA methylation, histone modification, histone variants, and ncRNAs [39]. Studies on *Brassica rapa* found that 127 differentially expressed lncRNAs were coexpressed with 128 differentially expressed genes, indicating that lncRNAs played an important role during vernalization [40]. In this study, 272 characterized lncRNAs were identified from *A. sinensis* and divided into six categories, namely (1) chromatin, DNA/RNA, and protein modification; (2) flowering; (3) stress response; (4) metabolism; (5) biosignaling; and (6) energy and transport, based on their coexpressed mRNAs.

FLC is a MADS-box transcriptional regulator that acts as a potent repressor of flowering [38]. In *Arabidopsis*, the epigenetic silencing of the floral repressor gene *FLC* is a well-characterized key event of vernalization [41]. In this study, 29 lncRNAs linked with chromatin, DNA/RNA, and protein modification were identified in *A. sinensis* during vernalization. For the chromatin modification, two coexpressed mRNAs, *HMGB2* and *HMGB3*, are involved in binding preferentially double-stranded DNA and up-regulated in response to cold stress [42]. For the DNA/RNA modification, *At1g05910* is involved in DNA demethylation and the negative regulation of chromatin silencing [43]; *RID2* is involved in rRNA methyltransferase activity [44]; and *At4g26600* is involved in RNA methylation [45]. For protein modification, 24 lncRNAs were involved and the roles of nine coexpressed mRNAs were represented as follows. *H2AV* plays a central role in regulating transcription, repairing DNA, replicating DNA, and stabilizing the nucleus chromosome [46]; *At2g28720* and *HTR2* are involved in compacting DNA into chromatin [47,48]; *HOS15* promotes the deacetylation of histone H4 [49]; *REF6* is involved in demethylating ‘Lys-27’ of histone H3, regulating flowering time by repressing *FLC* expression and interacting with the NF-Y complex to regulate SOC1 [50,51]; *SKP1A* and *ASK21* are involved in ubiquitination and form an SCF E3 ubiquitin ligase complex together with CUL1, RBX1, and an F-box protein [52,53]; and *SRK2G* and *SRK2H* are involved in protein phosphorylation [54,55]. In previous studies, *HMGB2* and *HMGB3* were up-regulated in response to cold stress but down-regulated in response to drought and salt stresses [42]; *H2AV*, *At2g28720* and *HTR2* exhibited a high level in response to osmotic and drought stresses [56]; *HOS15* was found to act as a repressor of cold stress-regulated gene expression and played a role in gene regulation for plant acclimation and tolerance to cold stress [49]; *SKP1A* and *ASK21* were overexpressed in the host stress response [57]; and *SRK2G* and *SRK2H* were found to be positive regulators in stress responses such as drought, salt, and cold [54,55,58]. Currently, although most of the lncRNAs have been reported to be involved in the stress response in many plants, their roles in the regulation of flowering time have been studied in model plants [23]. Previous studies have found that FLC in *Arabidopsis* is epigenetically regulated by lncRNAs *COOLAIR* and *COLDAIR* [59,60,61]. In this study, a lower expression level was noted for almost all coexpressed mRNAs of lncRNAs involved in chromatin, DNA/RNA, and protein modification at vernalization (T2, 0 °C) compared with freezing temperature (T3, −3 °C), as well as down-regulation at T2, indicating that these lncRNAs participate in epigenetic silencing by transferring euchromatin to heterochromatin and confer early bolting and flowering of *A. sinensis*. Representatively, the down-regulation of mRNAs *HMGB2* and *HMGB3* during vernalization transfers the heterochromatin to the euchromatin [42], which generates the ability of flowering; in contrast, their up-regulation at freezing temperatures inhibits flowering by keeping the heterochromatin, which indicates that their coexpressed lncRNAs play positive roles in regulating the flowering of *A. sinensis*. The down-regulation of mRNA *REF6* below 0 °C weakens the demethylation of histone H3 and delays flowering time by inducing FLC expression [50,51]; meanwhile, there is an increased expression level with decreased temperatures, which indicates that this coexpressed lncRNA plays a negative role in regulating the flowering of *A. sinensis*.

Flower formation occurs at the SAM and is a complex morphological event that is required not only for the circadian clock to measure the passage of time but also the regulation of meristem identity genes [42]. For the 6 representative coexpressed mRNAs of the 12 lncRNAs directly linked with flowering, *SRR1* is involved in a circadian clock input pathway and regulation of the expression of clock-regulated genes such as *CCA1* and *TOC1* [62]; *PHL* is involved in the circadian rhythm and the regulation of the timing of transition [63]; *PHYA* is involved in the regulation of flowering time and expression of its own gene as negative feedback [64]; *AGL62* is required for promoting the nuclear proliferation of early endosperm [65]; *AGL79* is involved in positively regulating the transition of the meristem from the vegetative to reproductive phase [66]; and *ATJ3* plays a continuous role in plant development, such as in photoperiodism, flowering, and positive regulation of flower development [67]. Previous studies found that mRNAs (e.g., miR156, miR169 and miR172) play a crucial role in developmental processes in rice, wheat, and maize, especially in the formation of the floral meristem, with miR172 controlling *AP2-like* genes [23,68,69]. Studies on the flowering of Chenopodium quinoa found that pivotal flowering homologs, including photoreceptor genes *PHYA* and *CRY1*, as well as genes associated with florigen-encoding genes (*FT* and *TWIN SISTER of FT*) and circadian clock-related genes (*ELF3*, *LHY*, and *HY5*), were specifically affected by night-break and competed with the positive- and negative-flowering lncRNAs [70]. In this study, down-regulation of all the coexpressed mRNAs involved in circadian clock and meristem identity genes was observed, which can be considered acceptable and reasonable, because these mRNAs are often highly expressed at the plant development stage (at photoperiod), while their expression levels were examined during vernalization. In addition, increased expression with decreased temperatures indicates that their coexpressed lncRNAs play negative roles in regulating the flowering of *A. sinensis*.

The expression of numerous lncRNAs has been demonstrated to be significantly affected by various stresses [23,30]. During vernalization, plants have to face and adapt to low temperature [38]. To date, extensive studies have reported that lncRNAs participate in defense responses associated with plant immunity and adaptation to the environment [22]. Heat-responsive lncRNAs have been found to be differentially expressed in *Brassica juncea*, and cold-responsive lncRNAs have been identified in grape and *Arabidopsis* [71,72,73]. lncRNAs could regulate HSP family genes (*HSP82* and *HSP83*) in response to heat stress in *Populus x canadensis* Moench, and *HSP18.1* in response to Cd stress *Betula platyphylla* [74,75]. For the eight representative coexpressed mRNAs of the 14 lncRNAs linked with the temperature response, *ACBP6* confers resistance to cold and freezing [76]; *ENO2* acts as a positive regulator in response to cold stress [77]; *ADH1* is required for survival and acclimation in response to abiotic stresses (e.g., cold, salt, and drought) [78,79]; *CSP2* contributes to the enhancement of cold and freezing tolerance [80]; and *HSP17.8*, *HSP70-3*, *HSP70-10*, and *HSP90-3* play vital roles in adapting to biotic and abiotic stresses [81,82]. In this study, increased expression for cold-tolerated mRNAs (*ACBP6*, *ENO2*, *ADH1*, and *CSP2*), and decreased expression for heat-tolerated mRNAs (*HSP17.8*, *HSP70-3*, *HSP70-10*, and *HSP90-3*), were observed with decreased temperatures, which indicates that their coexpressed lncRNAs play positive roles in adapting to low temperatures.

For vernalization to occur, sources of energy (sugars) and carbohydrate metabolism are required [37]. In recent years, the roles of lncRNAs in regulating metabolism in cancer, insulin, and chicken have been reported [83,84,85], while, in plants, studies are still limited. For the five representative coexpressed mRNAs of the 19 lncRNAs linked with carbohydrate metabolism, *CSY4* is involved in the synthesis of socitrate from oxaloacetate [86]; *GAPCP2* plays a critical role in glycolysis and exhibits up-regulation under drought stress [87,88]; *At3g52990* is involved in the synthesis of pyruvate from D-glyceraldehyde 3-phosphate [89]; *PGM2* participates in the synthesis of glucose [90]; and *BAM1* is required for starch breakdown [91]. In this study, the differential expression of these coexpressed mRNAs regulating sucrose and starch metabolism provided energy for the morphogenesis of seedlings and adaptation to low temperatures during vernalization. Representatively, the decreased expression of metabolite-synthesized mRNAs (*CSY4*, *At3g52990*, and *PGM2*), and increased expression of energy-produced mRNA *GAPCP2* and metabolite-degraded mRNA *BAM1,* were observed with decreased temperatures, which indicates that their coexpressed lncRNAs play positive roles in carbohydrate metabolism.

Endogenous hormones such as gibberellin, auxin, cytokinin, brassinosteroid and abscisic acid can either inhibit or promote flowering [37]. In previous studies, a pre-miRNA of miR393 was identified in *Brassica rapa* during vernalization, and the overexpression of an miR393-resistant form of TIR1 (mTIR1) could enhance auxin sensitivity, thus leading to pleiotropic effects on plant development [92]. For the 8 representative coexpressed mRNAs of the 13 lncRNAs linked with hormone signaling, *ARF1* is involved in the recruitment of COPI and GDAP1 to membranes and various auxin-dependent developmental processes [93]; *CUL1* participates in forming a SCF complex together with SKP1, RBX1, and a F-box protein and is involved in floral organ development, the auxin signaling pathway and ethylene signaling [94]; *SOFL4* and *SOFL5* are involved in cytokinin-mediated development [95]; *GRF2* and *GRF11* participate in the brassinosteroid (BR)-mediated signaling pathway [96,97]; *ERF3* is found to be differentially expressed in response to stresses and also regulates other ERFs [98]; and *SF1* is required for development and is involved in the alternative splicing of FLM pre-mRNA [99]. In this study, the differential expression of these mRNAs involved in hormone signaling also played certain roles in regulating the flower-bud differentiation of seedlings and cold tolerance during vernalization. In previous studies, *GRF2* and *ERF3* were found to be down-regulated, while *GRF11* was up-regulated in response to cold stress [100,101]; here, contrary findings for mRNAs *GRF2* and *GRF11* were observed with temperatures decreased, which indicate that their coexpressed lncRNAs may play negative roles in hormone signaling. The interaction between *SF1* and *FLM* pre-mRNA controls flowering time in response to temperature [102]; here, decreased expression of mRNA *SF1* was observed with decreased temperatures, which indicates that this coexpressed lncRNA may play a positive role in hormone signaling.

Energy generation and the transport of energy, nutrients and metabolites play essential roles in growth and development and stress tolerance [103]. For the 8 representative coexpressed mRNAs of the 43 lncRNAs linked with energy and transport, *PURU1* is involved in photorespiration and participates in preventing the excessive accumulation of 5-formyl tetrahydrofolate [104]; *MES16* is involved in chlorophyll breakdown by its demethylation [105]; *GPT1* is involved in NADPH generation through a series of processes including Glc6P transport, starch biosynthesis, fatty acid biosynthesis, and oxidative pentose phosphate [106]; *ABCF5* belongs to the ABC transporter superfamily and is involved in protein transport [107]; *SECA2* is involved in protein export coupling ATP hydrolysis [108]; *VPS26A* plays a role in vesicular protein sorting and is essential in endosome-to-Golgi retrograde transport [109]; *CML19* is a potential calcium sensor that binds calcium and is involved in the early response to stress [110]; and *NHX6* is involved in trafficking to the vacuole and exchanging the low-affinity electroneutral Na(+) or K(+)/H(+) [111]. In this study, the differential expression of these coexpressed mRNAs associated with energy and transport provided energy, nutrients, and metabolites for *A. sinensis* seedlings to obtain the capacity for vernalization and, meanwhile, to adapt to low temperatures.

**Figure 8:**
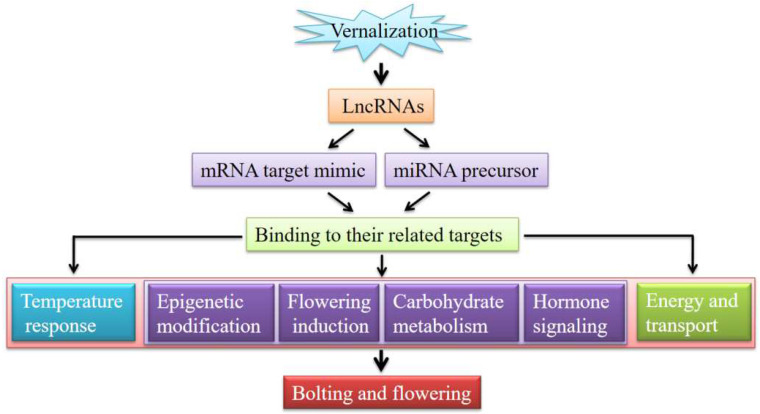
A proposed model of vernalization-induced bolting and flowering by regulatory lncRNAs in *A. sinensis*.

Based on the above functional analysis of lncRNAs identified in this study, flowering pathways proposed in the previous literature [14,15,16] and a general model of stress-responsive regulation by regulatory lncRNAs [23], a model of vernalization-induced bolting and flowering by regulatory lncRNAs in *A. sinensis* is proposed (Figure 8). Briefly, the vernalization of seedlings firstly triggers the differential expression of lncRNAs; then, the lncRNAs either act as a precursor of miRNAs or as a miRNA target mimic, which further binds their related targets; then, the binding of targets regulates the expression of their downstream mRNAs that are involved in various biological processes, including the temperature response, flowering pathways (i.e., epigenetic modification, flowering induction, carbohydrate metabolism, and hormone signaling), as well as energy and transport; finally, these biological processes promote the transition of the meristem from the vegetative phase to the bolting and flowering of *A. sinensis*.

## 5. Conclusions

From the above observations, we found that the lncRNAs positively or negatively regulated the expression of their downstream mRNAs through epigenetic changes at the level of transcription and post-transcription for the flowering of *A. sinensis* during vernalization. This coexpressed mRNA analysis of lncRNAs focused on five pathways, namely (1) chromatin, DNA/RNA, and protein modification; (2) floral development; (3) temperature response; (4) carbohydrate metabolism; and (5) hormone signaling. While several candidate lncRNAs were identified, their causative roles require further investigations.

## Figures and Tables

**Table 1:** Primer sequences used in qRT-PCR validation.

lncRNA ID	Coexpressed mRNAs	mRNA ID	Primer Sequences (5’ to 3’)	Amplicon Size (bp)
Isoform0062250	*HMGB2*	NM_001035997.1	Forward: CAAAGCTGCTGCTAAGGAC	155
			Reverse: GGACTTCCACTTGTCTCCAGC	
Isoform0061796	*HMGB3*	NM_001035998.1	Forward: CCTTCCAGTGCCTTCTTCGT	174
			Reverse: CTCAACCTTGCGCTTGTCAG	
Isoform0001498	*At1g05910*	NM_100472.2	Forward: AGACCACTCTCTCCGGTTGT	109
			Reverse: TCGTCAACTCCGATGACGTG	
Isoform0062769	*RID2*	NM_125110.6	Forward: GCAGGGCTTAGGTCTTCGTT	100
			Reverse: ACGAGGTTCATGCGATGACT	
Isoform0061049	*At4g26600*	NM_001341820.1	Forward: TTCCGATTGGTGCAACTCCT	118
			Reverse: GCCATGTCCACAACTCGTTC	
Isoform0034756	*H2AV*	NM_001339683.1	Forward: CAGTTGGACGAATTCACAGGC	176
			Reverse: CAGATGCCTTGGCGTTATCC	
Isoform0050517	*At2g28720*	NM_128433.4	Forward: GCAAGAAGCTTCAAAATTAGC	107
			Reverse: TGCTTAGCAAGTTCACCAGG	
Isoform0062503	*HTR2*	NM_113651.2	Forward: CACCGGAGGAGTGAAGAAGC	189
			Reverse: TCTTGAAGAGCTGCGACTGC	
Isoform0027210	*HOS15*	NM_126132.4	Forward: TACAGGCGCAGAACCTATGG	164
			Reverse: CTGTTGCATCACCAGACCCT	
Isoform0062048	*REF6*	NM_148863.4	Forward: AGGGAACACAGCTTCTGGTG	124
			Reverse: TTCCCCAAGTGAACGGTCTG	
Isoform0062818	*SKP1A*	NM_106245.5	Forward: GTGCTGCTACCTCCGATGAC	181
			Reverse: GTGCGGATCTCTTCTGGAGT	
Isoform0061474	*ASK21*	NM_001125404.1	Forward: CCTGATGACCTTACTGAGGAG	178
			Reverse: CAGGTCATCCACTGAACGCT	
Isoform0061497	*SRK2G*	NM_120946.5	Forward: ACATCGAGAGAGGTCGCAAG	110
			Reverse: AGGTGTCAGGATCACCTCCTT	
Isoform0062575	*SRK2H*	NM_125760.2	Forward: TGGTCCTGTGGTGTGACTCT	164
			Reverse: GAGAGAAGGTGTCTGCACTCC	
Isoform0062220	*SRR1*	NM_125348.4	Forward: ATCGCATTGTTTGGGAACAGC	117
			Reverse: AGCAAACTCGCTTGTGACTCT	
Isoform0061284	*PHL*	NM_001334526.1	Forward: CAAAGTCCTCGTTTGTCGGC	104
			Reverse: GCAACTGCTCCATAGTGGGT	
Isoform0057927	*PHYA*	NM_001123784.1	Forward: GTGCGATATGCTGATGCGTG	149
			Reverse: CCTGCAGGTGGAACTCACTT	
Isoform0041956	*AGL62*	NM_125437.5	Forward: CTCCTCACCAACACAACAAC	197
			Reverse: AACGCAAGTTCCTCAACGGG	
Isoform0045502	*AGL79*	NM_113925.3	Forward: AATCACCCCATGAGCTTCGC	107
			Reverse: TAGGGTTCCGGCAGCTACTT	
Isoform0063170	*ATJ3*	NM_114279.4	Forward: GAATACGCTCACGGAGTTGC	135
			Reverse: GCATCCCACTTGGCTCTCTC	
Isoform0062470	*ACBP6*	NM_102916.4	Forward: AATCACCCCATGAGCTTCGC	107
			Reverse: TAGGGTTCCGGCAGCTACTT	
Isoform0061783	*ENO2*	NM_129209.4	Forward: CACTGAGTGTGGAACCGAGG	190
			Reverse: GGTCATCACTCCCCAACCTG	
Isoform0063049	*ADH1*	NM_106362.3	Forward: TGTGACCGAGTGTGTGAACC	123
			Reverse: TGAATCATGGCCTGAACGCT	
Isoform0062198	*CSP2*	NM_120029.3	Forward: GATCTGGAGGTGGATACGGC	115
			Reverse: CAGTCTCTCGCCATGTGACC	
Isoform0062617	*HSP17.8*	NM_100614.3	Forward: AACATCGGCGATAACGAACG	154
			Reverse: CTCCACGTGTCTCTCTCCAC	
Isoform0009507	*HSP70-3*	NM_001202918.1	Forward: CGACTGCAGGAGACACTCAT	144
			Reverse: TCTCACAGGCGGTTCTCAAC	
Isoform0034676	*HSP70-10*	NM_120996.4	Forward: CGTGTCCCCAAGGTTCAGTC	167
			Reverse: CCGAGCGATAGAGGTGTGAC	
Isoform0042993	*HSP90-3*	NM_124983.4	Forward: AACAAGGAGGAGTACGCTGC	193
			Reverse: AGACACGACGGACATAGAGC	
Isoform0061974	*CSY4*	NM_001337082.1	Forward: GATGCAGAGCTCTACCGACC	197
			Reverse: CCTCTTCCGGGTCAAGCAAT	
Isoform0062375	*GAPCP2*	NM_101496.3	Forward: CATTTCTGCACCTTCAGCGG	198
			Reverse: TTCTGAGTAGCTGTGGTCGC	
Isoform0062268	*At3g52990*	NM_115159.5	Forward: GACAACTTGCGACCAACTCG	101
			Reverse: AATCCACGAAGGGTCTCAGC	
Isoform0062585	*PGM2*	NM_001160993.2	Forward: TGAACTGCGTACCCAAGGAG	158
			Reverse: TCGGTCTGCATCACCATCAG	
Isoform0062370	*BAM1*	NM_113297.3	Forward: AACTCTCTCGCTGTTCCTCG	165
			Reverse: GGAGAAGCCCGTCTCACAAT	
Isoform0062586	*ARF1*	NM_001337250.1	Forward: GTGACCGTGTTGTTGAAGCC	148
			Reverse: TGAAGCCCAAGCTTGTCAGT	
Isoform0061377	*CUL1*	NM_001036498.3	Forward: GTGCCGTGCATTGCTAAGAG	153
			Reverse: TCTTCGGCCTGTTGGACAAG	
Isoform0057235	*SOFL4*	NM_123240.2	Forward: AGGTCGTGGATGAGGACTAC	144
			Reverse: GAACCGCTGATAATTTGGCCC	
Isoform0043114	*SOFL5*	NM_001342234.1	Forward: TGCGAGTCAGGATGGACTCT	193
			Reverse: TCCTTGGACCAGAAGAAGCAT	
Isoform0062152	*GRF2*	NM_106479.3	Forward: AACTCTCCGGAATCTGCGAC	192
			Reverse: GAGCAGATTTGTAAGCGGCG	
Isoform0062828	*GRF11*	NM_001084180.2	Forward: GGTGCTAGGAGAGCATCGTG	198
			Reverse: GACGGTGGATTCTCCCGAAG	
Isoform0061395	*ERF3*	NM_103946.3	Forward: ATCGTTTAGCGGACCCAGAC	101
			Reverse: CGCAATCGCTGTGACAATCC	
Isoform0022533	*SF1*	NM_001036978.2	Forward: GGCTTAGGGTCAACTCCGAC	164
			Reverse: CCAGTCACACGGTCCTTGAT	
Isoform0044730	*PURU1*	NM_124115.4	Forward: ACGTCTTCTACTCTCGCAGC	125
			Reverse: AGGCACACGCACAACTGAAT	
Isoform0047216	*MES16*	NM_117770.5	Forward: CCATCCCTTCTCCGCATCTT	195
			Reverse: TCATAGGAGCAGGACGCAAC	
Isoform0063248	*GPT1*	NM_124861.5	Forward: CGCTGGTTCGTTGATGATGC	193
			Reverse: AAACGCAGGTTCACCACTCT	
Isoform0062571	*ABCF5*	NM_125882.3	Forward: TGCTGATAGGCTTGTGGCTT	103
			Reverse: CGGCTCATCAAGTAGCAGCA	
Isoform0008194	*SECA2*	NM_001198130.1	Forward: ACTGTGAGGCCCATTGTCTG	117
			Reverse: CTCTGCCACGAAGCTGGTTA	
Isoform0062373	*VPS26A*	NM_124733.4	Forward: TGTTCCGCTTCCTCCAATCAA	196
			Reverse: TGCTCCAGTTGATTCTCGCC	
Isoform0062449	*CML19*	NM_119864.5	Forward: CGAGCTCAACGTTGCTATGAG	160
			Reverse: GTCTATGGAGTCTCGTTCTCCG	
Isoform0061307	*NHX6*	NM_106609.4	Forward: GGCATTTGCTCTTGCTCTGC	112
			Reverse: TCCTCCAATCAGCAACACCG	

**Table 2:** Twenty-nine lncRNAs linked with chromatin, DNA/RNA, and protein modification.

lncRNA ID	Coexpressed mRNAs	mRNA ID	Proteins Encoded by Coexpressed mRNAs
**Chromatin modification (2)**			
Isoform0062250	*HMGB2*	AT1G20693.1	High mobility group B protein 2
Isoform0061796	*HMGB3*	AT1G20696.1	High mobility group B protein 3
**DNA/RNA modification (3)**			
Isoform0001498	*At1g05910*	AT1G05910.1	ATPase family AAA domain-containing protein At1g05910
Isoform0062769	*RID2*	AT5G57280.1	18S rRNA (guanine-N(7))-methyltransferase RID2 e
Isoform0061049	*At4g26600*	AT4G26600.8	S-adenosyl-L-methionine-dependent methyltransferases superfamily protein
**Protein modification (24)**			
Isoform0034756	*H2AV*	AT3G54560.1	Histone H2A variant 1
Isoform0050517	*At2g28720*	AT2G28720.1	Histone H2B.3
Isoform0062503	*HTR2*	AT1G09200.1	Histone H3.2
Isoform0027210	*HOS15*	AT5G67320.1	WD40 repeat-containing protein HOS15
Isoform0062048	*REF6*	AT3G48430.1	Lysine-specific demethylase REF6
Isoform0062818	*SKP1A*	AT1G75950.1	SKP1-like protein 1A
Isoform0061474	*ASK21*	AT3G61415.1	SKP1-like protein 21
Isoform0061497	*SRK2G*	AT5G08590.1	Serine/threonine-protein kinase SRK2G
Isoform0062575	*SRK2H*	AT5G63650.1	Serine/threonine-protein kinase SRK2H
Isoform0053138	*DET1*	AT4G10180.1	Light-mediated development protein DET1
Isoform0030135	*BOPAt4g295601*	AT3G57130.2	Ankyrin repeat family protein/BTB/POZ domain-containing protein
Isoform0063715	*At1g45180*	AT1G45180.1	F27F5.26
Isoform0044146	*At3g50840*	AT3G50840.3	Phototropic-responsive NPH3 family protein
Isoform0034163	*At2g36630*	AT2G36630.1	Sulfite exporter TauE/SafE family protein 4
Isoform0058941	*At3g47890*	AT3G47890.2	Ubiquitin carboxyl-terminal hydrolase-related protein
Isoform0007659	*UBP7*	A0A1I9LL79	Ubiquitin carboxyl-terminal hydrolase
Isoform0045576	*DER2.1*	AT3G21280.2	Derlin-2.1
Isoform0062510	*GRP3*	AT2G05520.1	Glycine-rich protein 3
Isoform0062575	*MDH9.13*	AT5G42440.1	At5g42440
Isoform0051826	*At3g24715*	AT3G24715.1	Kinase superfamily with octicosapeptide/Phox/Bem1p domain-containing protein
Isoform0061548	*At3g16560*	AT3G16560.4	Probable protein phosphatase 2C 40
Isoform0049189	*At3g62260*	AT3G62260.1	Probable protein phosphatase 2C 49
Isoform0060667	*ESMD1*	AT2G01480.1	Protein ESMERALDA 1
Isoform0062775	*At1g27930*	AT1G27930.1	Probable methyltransferase At1g27930

**Table 3:** Twelve lncRNAs directly linked with flowering.

lncRNA ID	Coexpressed mRNAs	mRNA ID	Proteins Encoded by Coexpressed mRNAs
Isoform0062220	*SRR1*	AT5G59560.2	Protein SENSITIVITY TO RED LIGHT REDUCED 1
Isoform0061284	*PHL*	AT1G72390.1	Protein PHYTOCHROME-DEPENDENT LATE-FLOWERING
Isoform0057927	*PHYA*	AT1G09570.6	Phytochrome A
Isoform0041956	*AGL62*	AT5G60440.1	Agamous-like MADS-box protein AGL62
Isoform0045502	*AGL79*	AT3G30260.1	AGAMOUS-like 79
Isoform0063170	*ATJ3*	AT3G44110.1	Chaperone protein dnaJ 3
Isoform0028325	*BBX29*	AT5G54470.1	At5g54470
Isoform0054894	*CLE13*	AT1G73965.1	CLAVATA3/ESR (CLE)-related protein 13
Isoform0061298	*CLE44*	AT4G13195.1	CLAVATA3/ESR (CLE)-related protein 44
Isoform0056751	*MXC17.10*	AT5G24710.1	Transducin/WD40 repeatlike superfamily protein
Isoform0062524	*At1g06515*	AT1G06515.2	Transmembrane protein, putative (DUF3317)
Isoform0061573	*BHLH30*	AT1G68810.1	Transcription factor bHLH30

**Table 4:** Fourteen lncRNAs directly linked with temperature response.

lncRNA ID	Coexpressed mRNAs	mRNA ID	Proteins Encoded by Coexpressed mRNAs
Isoform0062470	*ACBP6*	AT1G31812.1	Acyl-CoA-binding domain-containing protein 6
Isoform0061783	*ENO2*	AT2G36530.1	Bifunctional enolase 2/transcriptional activator
Isoform0063049	*ADH1*	AT1G77120.1	Alcohol dehydrogenase class-P
Isoform0062198	*CSP2*	AT4G38680.1	Cold shock protein 2
Isoform0035932	*RH20*	AT1G55150.2	DEA(D/H)-box RNA helicase family protein
Isoform0019851	*RH52*	AT3G58570.1	DEAD-box ATP-dependent RNA helicase 52
Isoform0062484	*RH53*	AT3G22330.1	DEAD-box ATP-dependent RNA helicase 53, mitochondrial
Isoform0058095	*RAB18*	AT5G66400.1	Dehydrin Rab18
Isoform0061968	*XERO1*	AT3G50980.1	Dehydrin Xero 1
Isoform0020919	*MED14*	AT3G04740.1	Mediator of RNA polymerase II transcription subunit 14
Isoform0062617	*HSP17.8*	AT1G07400.1	17.8 kDa class I heat shock protein
Isoform0009507	*HSP70-3*	AT3G09440.2	Heat shock 70 kDa protein 3
Isoform0034676	*HSP70-10*	AT5G09590.1	Heat shock 70 kDa protein 10, mitochondrial
Isoform0042993	*HSP90-3*	AT5G56010.1	Heat shock protein 90-3

**Table 5:** Nineteen lncRNAs directly linked with carbohydrate metabolism.

lncRNA ID	Coexpressed mRNAs	mRNA ID	Proteins Encoded by Coexpressed mRNAs
Isoform0061974	*CSY4*	AT2G44350.2	Citrate synthase 4, mitochondrial
Isoform0062251	*FBA3*	AT2G01140.1	Fructose-bisphosphate aldolase 3, chloroplastic
Isoform0062131	*GAPC1*	AT3G04120.1	Glyceraldehyde-3-phosphate dehydrogenase GAPC1, cytosolic
Isoform0062375	*GAPCP2*	AT1G16300.1	Glyceraldehyde-3-phosphate dehydrogenase GAPCP2, chloroplastic
Isoform0062268	*At3g52990*	AT3G52990.1	Pyruvate kinase
Isoform0062585	*PGM2*	AT1G70730.3	Phosphoglucomutase (alpha-D-glucose-1,6-bisphosphate-dependent)
Isoform0063034	*USP*	AT5G52560.1	UDP-sugar pyrophosphorylase
Isoform0042435	*GLCNAC1PUT2*	AT2G35020.1	UDP-N-acetylglucosamine diphosphorylase 2
Isoform0062018	*UXS2*	AT3G62830.1	UDP-glucuronic acid decarboxylase 2
Isoform0062750	*XYLA*	AT5G57655.2	Xylose isomerase
Isoform0060700	*GALS1*	AT2G33570.1	Galactan beta-1,4-galactosyltransferase GALS1
Isoform0062032	*OFUT31*	AT4G24530.1	O-fucosyltransferase 31
Isoform0039872	*At5g67460*	AT5G67460.1	Glucan endo-1,3-beta-D-glucosidase
Isoform0045893	*RSW3*	AT5G63840.2	Glycosyl hydrolases family 31 protein
Isoform0036200	*At1g59950*	AT1G59950.1	Aldo/keto reductase
Isoform0042053	*At5g25970*	AT5G25970.2	Core-2/I-branching beta-1,6-N-acetylglucosaminyl-transferase family protein
Isoform0059181	*UGT76E7*	AT5G38040.1	UDP-glycosyltransferase 76E7
Isoform0037698	*At1g26850*	AT1G26850.2	Probable methyltransferase PMT2
Isoform0062370	*BAM1*	AT3G23920.1	Beta-amylase 1, chloroplastic

**Table 6:** Thirteen lncRNAs directly linked with hormone signaling.

lncRNA ID	Coexpressed mRNAs	mRNA ID	Proteins Encoded by Coexpressed mRNAs
Isoform0062586	*ARF1*	AT2G47170.1	ADP-ribosylation factor 1
Isoform0061377	*CUL1*	AT4G02570.1	Cullin-1
Isoform0015752	*T4L20.330*	AT4G34750.1	SAUR-like auxin-responsive protein family
Isoform0057235	*SOFL4*	AT5G38790.1	Protein SOB FIVE-LIKE 4
Isoform0043114	*SOFL5*	AT4G33800.2	Protein SOB FIVE-LIKE 5
Isoform0062152	*GRF2*	AT1G78300.1	14-3-3-like protein GF14 omega
Isoform0062828	*GRF11*	AT1G34760.1	14-3-3-like protein GF14 omicron
Isoform0061395	*ERF3*	AT1G50640.1	Ethylene-responsive transcription factor 3
Isoform0007735	*CIPK20*	AT5G45820.1	CBL-interacting serine/threonine-protein kinase 20
Isoform0022533	*SF1*	AT5G51300.2	Splicing factorlike protein 1
Isoform0046311	*AGD9*	AT5G46750.1	Probable ADP-ribosylation factor GTPase-activating protein AGD9
Isoform0057859	*TIFY4B*	AT4G14720.1	Protein TIFY 4B
Isoform0062437	*At2g34810*	AT2G34810.1	Berberine bridge enzyme-like 16

**Table 7:** Forty-three lncRNAs linked with energy and transport.

lncRNA ID	Coexpressed mRNAs	mRNA ID	Proteins Encoded by Coexpressed mRNAs
**Energy (5)**			
Isoform0044730	*PURU1*	AT5G47435.2	Formyltetrahydrofolate deformylase 1, mitochondrial
Isoform0031698	*ndhB1*	ATCG01250.1	NAD(P)H-quinone oxidoreductase subunit 2 A, chloroplastic
Isoform0029144	*NDB1*	AT4G28220.2	NADH:ubiquinone reductase (nonelectrogenic)
Isoform0046995	*WNK9*	AT5G28080.3	Nonspecific serine/threonine protein kinase
Isoform0047216	*MES16*	AT4G16690.1	pFDCC methylesterase MES16
**Transport (38)**			
Isoform0047603	*At5g11230*	AT5G11230.1	Probable sugar phosphate/phosphate translocator At5g11230
Isoform0063248	*GPT1*	AT5G54800.1	Glucose-6-phosphate/phosphate translocator 1, chloroplastic
Isoform0053163	*SMXL5*	AT5G57130.1	Protein SMAX1-LIKE 5
Isoform0062571	*ABCF5*	AT5G64840.1	ABC transporter F family member 5
Isoform0062182	*SFH8*	AT2G21520.2	Phosphatidylinositol/phosphatidylcholine transfer protein SFH8
Isoform0022607	*BASS6*	AT4G22840.1	Probable sodium/metabolite cotransporter BASS6, chloroplastic
Isoform0008194	*SECA2*	AT1G21650.3	Protein translocase subunit SECA2, chloroplastic
Isoform0014041	*At4g14160*	AT4G14160.1	Protein transport protein SEC23
Isoform0062660	*ycf2-A*	ATCG01280.1	Protein Ycf2
Isoform0036773	*At4g22990*	AT4G22990.1	SPX domain-containing membrane protein At4g22990
Isoform0032676	*At3g49350*	AT3G49350.1	At3g49350
Isoform0062443	*TMT2*	AT4G35300.5	Tonoplast monosaccharide transporter2
Isoform0003274	*VPS24-1*	AT5G22950.1	Vacuolar protein sorting-associated protein 24 homolog 1
Isoform0062373	*VPS26A*	AT5G53530.1	Vacuolar protein sorting-associated protein 26A
Isoform0028575	*VPS52*	AT1G71270.1	Vacuolar protein sorting-associated protein 52 A
Isoform0038654	*VPS60-2*	AT5G04850.1	Vacuolar protein sorting-associated protein 60.2
Isoform0062996	*At5g19500*	AT5G19500.1	At5g19500
Isoform0062800	*PIP1-5*	AT4G23400.1	Probable aquaporin PIP1-5
Isoform0061992	*SULTR2;2*	AT1G77990.1	Sulfate transporter 2.2
Isoform0062606	*MT2A*	AT3G09390.2	Metallothionein-like protein 2A
Isoform0057136	*At3g52300*	AT3G52300.1	ATP synthase subunit d, mitochondrial
Isoform0061450	*ABCB23*	AT4G28630.1	ABC transporter B family member 23, mitochondrial
Isoform0062449	*CML19*	AT4G37010.1	Calcium-binding protein CML19
Isoform0061763	*JJJ1*	AT1G74250.1	DNAJ protein JJJ1 homolog
Isoform0061955	*PAP1*	AT1G13750.1	Probable inactive purple acid phosphatase 1
Isoform0062074	*HIPP04*	AT1G29000.2	Heavy metal-associated isoprenylated plant protein 4
Isoform0062972	*HIPP26*	AT4G38580.1	Heavy metal-associated isoprenylated plant protein 26
Isoform0014712	*KINUA*	AT1G12430.1	Kinesin-like protein KIN-UA
Isoform0063051	*IQD30*	AT1G18840.2	Protein IQ-DOMAIN 30
Isoform0016777	*At3g18430*	AT3G18430.1	Calcineurin b subunit (Protein phosphatase 2b regulatory subunit)-like protein
Isoform0063921	*CBL10*	AT4G33000.6	Calcineurin B-like protein 10
Isoform0061307	*NHX6*	AT1G79610.1	Sodium/hydrogen exchanger 6
Isoform0024682	*CNGC15*	AT2G28260.2	Cyclic nucleotide-gated channel 15
Isoform0034638	*2-Oct*	AT1G79360.1	Organic cation/carnitine transporter 2
Isoform0033810	*CNGC13*	AT4G01010.2	Putative cyclic nucleotide-gated ion channel 13
Isoform0051075	*MTG13.4*	AT5G16680.1	RING/FYVE/PHD zinc finger superfamily protein
Isoform0051563	*SUF4*	AT1G30970.3	Zinc finger (C2H2 type) family protein
Isoform0037483	*AHA4*	AT3G47950.2	ATPase 4, plasma membranetype

## Data Availability

The datasets are available at https://www.ncbi.nlm.nih.gov/bioproject/PRJNA789039 (release date on 13 January 2022).

## Supplementary Materials

The following supporting information can be downloaded at: https://www.mdpi.com/article/10.3390/cimb44050128/s1.

## Abbreviations

AP1	APETALA 1
CCS	Circular consensus sequence
CNCI	Coding–Noncoding Index
COLDAIR	COLD-ASSISTED INTRONIC NON-CODING RNA
COOLAIR	COLD-INDUCED LONG ANTISENSE INTRAGENIC RNAs
CPC	Coding-Potential Calculator
FLC	FLOWERING LOCUS C
FLM	FLOWERING LOCUS M
FLNC	Full-length nonchimeric
FT	FLOWERING LOCUS T
GA	Gibberellin
GA2OX1	Gibberellin 2-β-dioxygenase 1
GO	Gene Ontology
KEGG	Kyoto Encyclopedia of Genes and Genomes
KOG	euKaryotic orthologous groups of proteins
lncRNAs	Long noncoding RNAs
NCBI	National Center for Biotechnology Information
ncRNAs	Noncoding RNAs
NR	NCBI nonredundant protein
PHYA	PHYOCHROME A
REL	Relative expression level
SOC1	SUPPRESSOR OF OVEREXPRESSION OF CONSTANS 1
sRNAs	Small RNAs
TAIR	The Arabidopsis Information Resource
