# Supplementary material for: Transcriptomic Analysis Reveals LncRNAs Associated with Flowering of Angelica sinensis during Vernalization
Source: Curr Issues Mol Biol. 2022 Apr 26;44(5):1867–88. doi: 10.3390/cimb44050128 (PMC9164074; doi:10.3390/cimb44050128)
Supplement: Supplementary file 1 [file cimb-44-00128-s001.zip › Supplementary Materials.pdf]

Figure supplementary legends

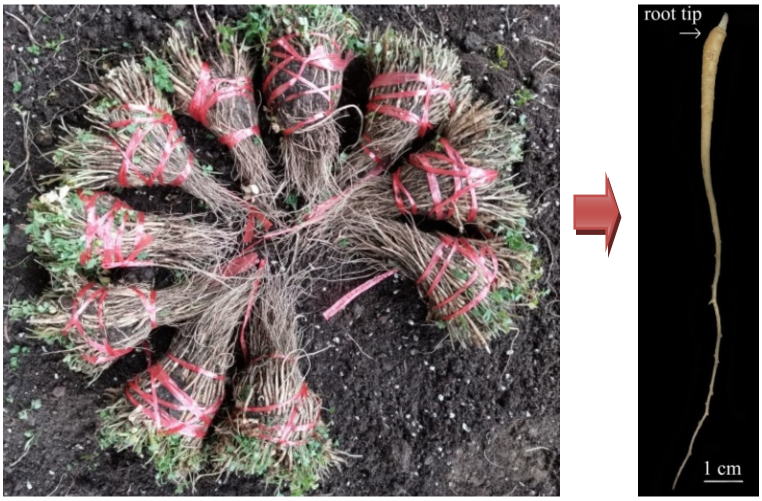

Figure S1. Morphological characteristic of *Angelica sinensis* seedlings.

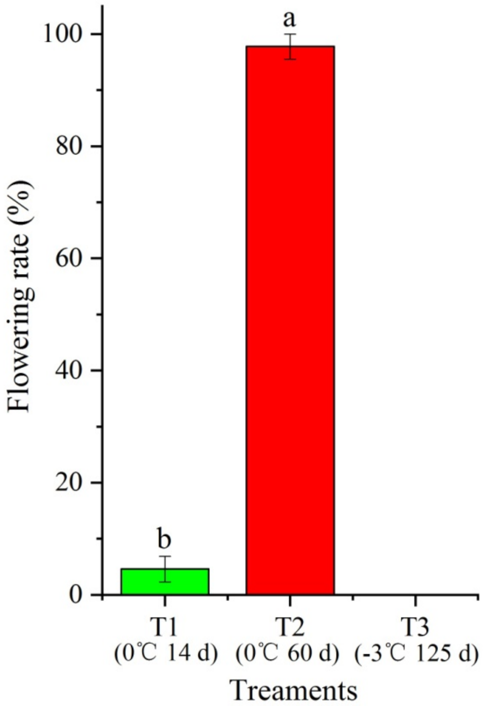

Figure S2. Flowering rate of *A. sinensis* after the seedlings stored at T1, T2 and T3. Different letters represents a significant difference ($P < 0.05$) at different treatments.

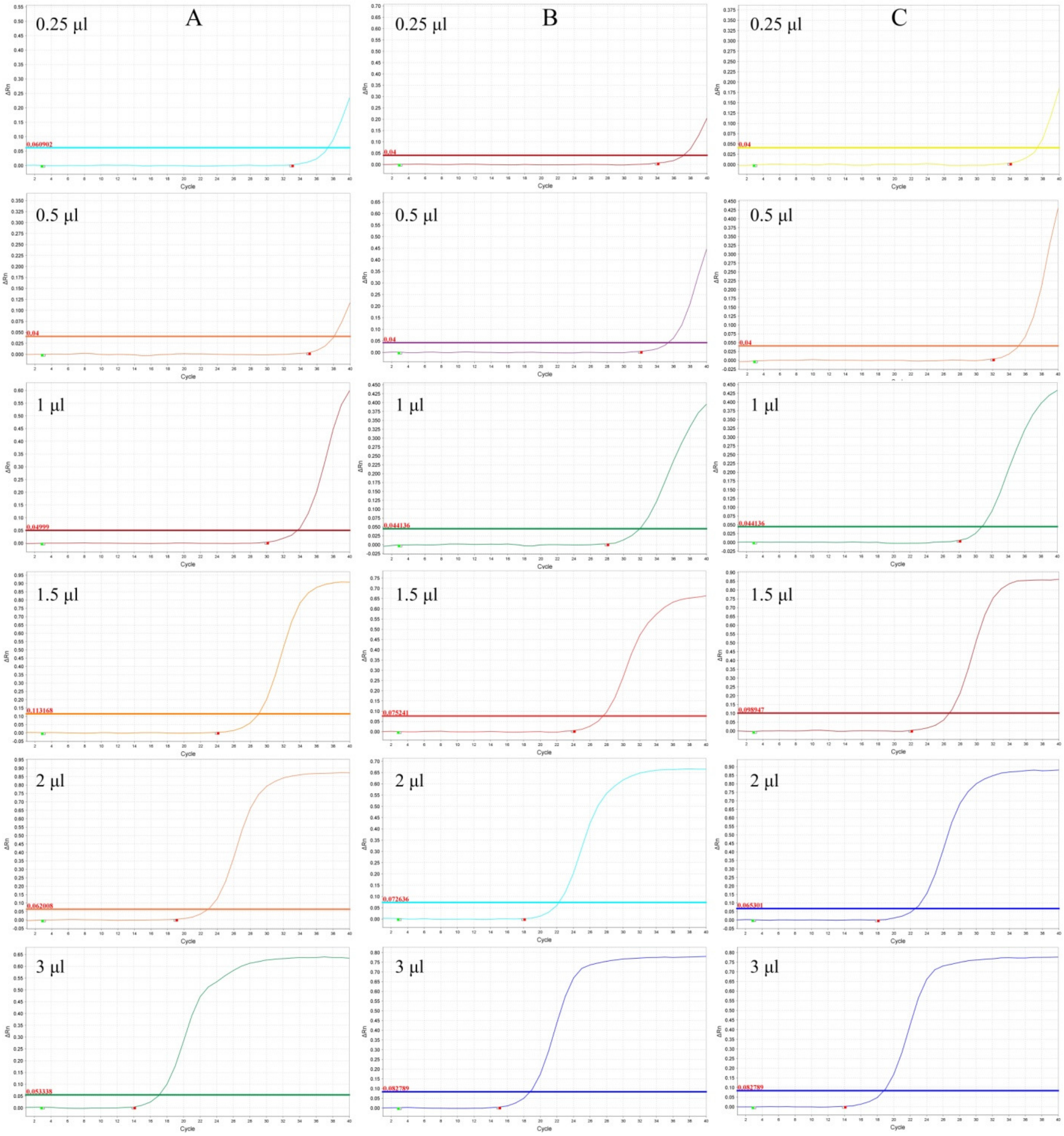

Figure S3. The cycle threshold (Ct) values of *ACT* gene at different volumes (0.25, 0.5, 1.0, 1.5, 2.0 and 3.0 µL) via PCR amplification with three replications.

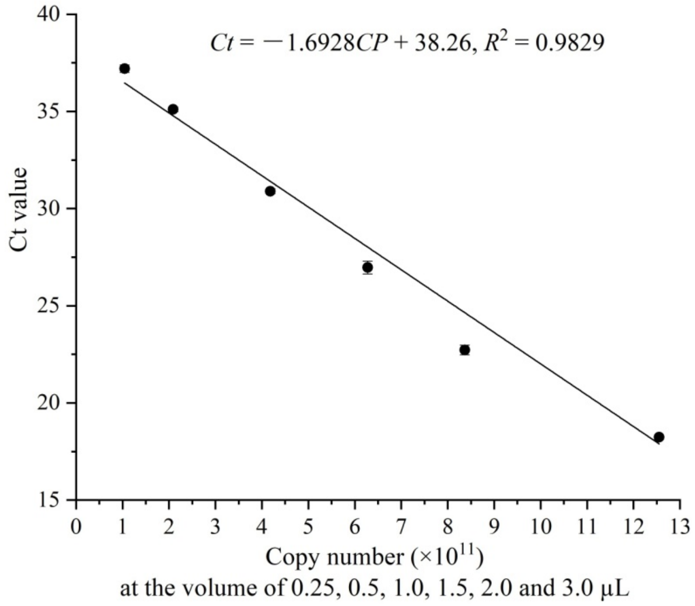

Figure S4. The standard curve of *ACT* gene.

Table supplementary legends

Table S1. The expression level of the 49 representative genes and their standard deviations for every variant. Note, the Table S1 is shown as a document of Excel format.

Table S2. The base sequences of the 272 lncRNAs identified from *A. sinensis*.. Note, the Table S2 is shown as a document of Excel format.

Table S3. Twenty-four lncRNAs indirectly involved in flowering.

LncRNAs ID	Co-expressed mRNAs	mRNA ID	Proteins Encoded by Co-expressed mRNAs
Isoform0061916	*NOF1*	AT1G17690.1	U3 small nucleolar RNA-associated protein
Isoform0031076	*XPO1B*	AT3G03110.1	Protein EXPORTIN 1B
Isoform0007968	*ARL8B*	AT5G67560.1	ADP-ribosylation factor-like protein 8b
Isoform0063120	*KRP3*	AT5G48820.1	Cyclin-dependent kinase inhibitor 3
Isoform0061684	*CYCU4-3*	AT5G61650.1	Cyclin-U4-3
Isoform0062002	*At5g06110*	AT5G06110.2	Cell division related protein-like
Isoform0044000	*KTN80.2*	AT1G61210.3	Katanin p80 WD40 repeat-containing subunit B1 homolog KTN80.2
Isoform0062298	*KIN12B*	AT3G23670.1	Kinesin-like protein KIN-12B
Isoform0062035	*F14P22.240*	AT3G58650.1	GPI-anchored adhesin-like protein
Isoform0057493	*SCD2*	AT3G48860.3	Coiled-coil protein
Isoform0062436	*ASY1*	AT1G67370.1	Meiosis-specific protein ASY1
Isoform0038510	*AMC5*	AT1G79330.1	Metacaspase-5
Isoform0025252	*AGP18*	AT4G37450.1	Lysine-rich arabinogalactan protein 18
Isoform0060185	*WIH2*	AT2G41420.1	Cysteine-rich and transmembrane domain-containing protein WIH2
Isoform0061173	*EPR1*	AT2G27380.1	Proline-rich extensin-like protein EPR1
Isoform0057289	*LRX2*	AT1G62440.1	Leucine-rich repeat extensin-like protein 2
Isoform0062989	*At5g51670*	AT5G51670.1	Similarity to unknown protein
Isoform0007953	*TON2*	AT5G18580.1	Probable serine/threonine-protein phosphatase 2A regulatory subunit B'' subunit TON2
Isoform0062133	*PRF3*	AT5G56600.1	Profilin-3
Isoform0010206	*TUBA4*	AT1G04820.1	Tubulin alpha-4 chain
Isoform0062730	*TUBB5*	AT1G20010.1	Tubulin beta-5 chain
Isoform0061345	*At5g04460*	AT5G04460.1	RING/U-box superfamily protein
Isoform0061501	*PMEI10*	AT1G62760.1	Pectinesterase inhibitor 10
Isoform0037378	*SBT1.6*	AT4G34980.1	Subtilisin-like protease SBT1.6

Table S4. Ten lncRNAs involved in other stresses response.

LncRNAs ID	Co-expressed mRNAs	mRNA ID	Proteins Encoded by Co-expressed mRNAs
Isoform0062501	*MUA22.11*	AT5G14110.1	Peroxidase (DUF 3339)
Isoform0062206	*GPX6*	AT4G11600.1	Probable phospholipid hydroperoxide glutathione peroxidase 6, mitochondrial
Isoform0061942	*ERD10*	AT1G20450.2	Dehydrin family protein
Isoform0062277	*F6I1.15*	AT1G16850.1	At1g16880
Isoform0044679	*At1g62480*	AT1G62480.1	At1g62480/T3P18_4
Isoform0053250	*At1g70870*	AT1G70870.1	Polyketide cyclase/dehydrase and lipid transport superfamily protein
Isoform0050062	*At1g13608*	AT1G13608.1	Putative defensin-like protein 288
Isoform0062612	*RTNLB1*	AT4G23630.2	Reticulon-like protein B1

Isoform0062386	*SDF2*	AT2G25110.1	Stromal cell-derived factor 2-like protein
Isoform0062893	*NBR1*	AT4G24690.1	Protein NBR1 homolog

Table S5. Ninety-eight lncRNAs involved in nucleotide, protein, amino acid and lipid metabolism.

LncRNAs ID	Co-expressed mRNAs	mRNA ID	Proteins Encoded by Co-expressed mRNA
**Nucleotide metabolism (38)**			
Isoform0062467	*CBP60B*	AT5G57580.1	Calmodulin-binding protein 60 B
Isoform0062053	*CBP60C*	AT2G18750.3	Calmodulin-binding protein 60 C
Isoform0033248	*IDD5*	AT2G02070.2	Protein indeterminate-domain 5, chloroplastic
Isoform0063322	*P1R1*	AT5G57780.1	At5g57780
Isoform0062703	*emb1579*	AT2G03150.2	ATP/GTP-binding protein family
Isoform0021844	*Negative on TATA less2a*	AT1G07705.1	NOT2 / NOT3 / NOT5 family
Isoform0051920	*At5g15040*	AT5G15040.1	Paired amphipathic helix (PAH2) superfamily protein
Isoform0061917	*At5g25590*	AT5G25590.1	DNA ligase (DUF630 and DUF632)
Isoform0062672	*At4g31150*	AT4G31150.3	Endonuclease V family protein
Isoform0030117	*At4g29560*	AT4G29560.1	Fanconi anemia group E protein FANCE protein
Isoform0045387	*At1g52500*	AT1G52500.5	DNA-(apurinic or apyrimidinic site) lyase
Isoform0032925	*At1g54440*	AT1G54440.4	Polynucleotidyl transferase, ribonuclease H fold protein with HRDC domain-containing protein
Isoform0062838	*F8A24.10*	AT3G09850.1	AT3g09850/F8A24_10
Isoform0048086	*NOP10*	AT2G20490.2	Nucleolar protein 10
Isoform0013277	*NUDT17*	AT2G01670.1	Nudix hydrolase 17, mitochondrial
Isoform0029722	*CDA1*	AT2G19570.1	Cytidine deaminase 1
Isoform0062946	*POLD1*	AT5G63960.2	DNA polymerase delta catalytic subunit
Isoform0062489	*APT5*	AT5G11160.2	Adenine phosphoribosyltransferase
Isoform0055697	*AT1G53040.1*	AT1G53040.1	Putative tRNA (Met) cytidine acetyltransferase
Isoform0061427	*GFL*	AT5G25230.1	109 kDa U5 small nuclear ribonucleoprotein component GFL
Isoform0063253	*CRS2A*	AT5G38290.1	Chloroplastic group IIB intron splicing facilitator CRS2-A, chloroplastic
Isoform0061484	*RNP1*	AT4G14300.1	Heterogeneous nuclear ribonucleoprotein 1
Isoform0059903	*SR34B*	AT4G02430.1	Serine/arginine-rich splicing factor SR34B
Isoform0063219	*At4g17410*	AT4G17410.4	DWNN domain, a CCHC-type zinc finger
Isoform0060560	*NUCL1*	AT1G48920.1	Nucleolin 1
Isoform0062273	*EMB140*	AT4G24270.3	EMBRYO DEFECTIVE 140
Isoform0061426	*GR-RBP2*	AT4G13850.3	Glycine-rich RNA-binding protein 2
Isoform0060947	*HSFC1*	AT3G24520.1	Heat stress transcription factor C-1
Isoform0062360	*At5g46030*	AT5G46030.1	Transcription elongation factor 1 homolog
Isoform0061214	*TAF15B*	AT5G58470.1	Transcription initiation factor TFIID subunit 15b
Isoform0062411	*At2g25670*	AT2G25670.1	Expressed protein
Isoform0050784	*NUP54*	AT1G24310.1	Nuclear pore complex protein NUP54
Isoform0048863	*MOS11*	AT5G02770.1	Protein MODIFIER OF SNC1 11
Isoform0062166	*ALY2*	AT5G02530.1	THO complex subunit 4B
Isoform0039454	*PCMP-E29*	AT3G21470.1	Pentatricopeptide repeat-containing protein At3g21470
Isoform0062860	*At2g44120*	AT2G44120.2	Ribosomal protein L30/L7 family protein
Isoform0045623	*At2g01640*	AT2G01640.2	Expressed protein
Isoform0063106	*At1g69070*	AT1G69070.2	Nucleolar-like protein
**Protein, amino acid and lipid metabolism (55)**			

Isoform0055609	*rps3*	ATCG00800.1	30S ribosomal protein S3, chloroplastic
Isoform0019098	*rps11*	ATCG00750.1	30S ribosomal protein S11, chloroplastic
Isoform0062529	*RPS17D*	AT5G04800.2	40S ribosomal protein S17-4
Isoform0062975	*RPS28A*	AT5G03850.1	40S ribosomal protein S28-1
Isoform0062903	*At2g27720*	AT2G27720.4	60S acidic ribosomal protein family
Isoform0062942	*RPL8C*	AT4G36130.1	60S ribosomal protein L8-3
Isoform0062966	*RPL27A*	AT2G32220.1	60S ribosomal protein L27-1
Isoform0062415	*RPL32B*	AT5G46430.1	60S ribosomal protein L32-2
Isoform0062322	*RPL41A*	AT3G08520.1	60S ribosomal protein L41
Isoform0062695	*RPP1C*	AT5G47700.1	60S acidic ribosomal protein P1-3
Isoform0063056	*RPP2D*	AT3G44590.2	60S acidic ribosomal protein P2-4
Isoform0062650	*A4*	AT5G60390.1	Elongation factor 1-alpha 4
Isoform0062070	*EIF3B-2*	AT5G25780.1	Eukaryotic translation initiation factor 3 subunit B
Isoform0063115	*At4g27130*	AT4G27130.1	Protein translation factor SUI1 homolog 1
Isoform0062691	*At1g44770*	AT1G44770.1	Submitted name: At1g44770
Isoform0062400	*ELF5A-3*	AT1G69410.1	Eukaryotic translation initiation factor 5A-3
Isoform0047687	*At5g05990*	AT5G05990.1	AT5g05990/K18J17_19
Isoform0062112	*CRT1*	AT1G56340.1	Calreticulin-1
Isoform0048497	*CRT3*	AT1G08450.1	Calreticulin-3
Isoform0062371	*LON2*	AT5G47040.1	Lon protease homolog 2, peroxisomal
Isoform0045559	*CYP63*	AT3G63400.2	Peptidyl-prolyl cis-trans isomerase CYP63
Isoform0042613	*FKBP15-1*	AT3G25220.1	Peptidyl-prolyl cis-trans isomerase FKBP15-1
Isoform0062632	*CCT5*	AT1G24510.1	T-complex protein 1 subunit epsilon
Isoform0062621	*At3g12390*	AT3G12390.1	Nascent polypeptide-associated complex subunit a-like protein 1
Isoform0043033	*MXH1.2*	AT5G35680.1	Eukaryotic translation initiation factor 4C
Isoform0061282	*ERF1-3*	AT3G26618.1	Eukaryotic peptide chain release factor subunit 1-3
Isoform0062390	*F21O3.19*	AT3G07480.1	2Fe-2S ferredoxin-like superfamily protein
Isoform0062110	*At5g18400*	AT5G18400.1	Anamorsin homolog
Isoform0028159	*At5g15570*	AT5G15570.1	BTP domain-containing protein
Isoform0061623	*BEE1*	AT1G18400.2	BR enhanced expression 1
Isoform0063558	*FACE1*	AT4G01320.1	CAAX prenyl protease 1 homolog
Isoform0061264	*ATE1*	AT5G05700.2	Arginyl-tRNA--protein transferase
Isoform0053818	*At5g16090*	AT5G16090.1	Ubiquitin receptor RAD23
Isoform0007860	*At2g27420*	AT2G27420.1	Cysteine proteinase
Isoform0061074	*CYOP*	AT5G10540.1	Probable cytosolic oligopeptidase A
Isoform0063176	*At2g39710*	AT2G39710.1	Eukaryotic aspartyl protease family protein
Isoform0062190	*At2g41250*	AT2G41250.1	Haloacid dehalogenase-like hydrolase (HAD) superfamily protein
Isoform0006225	*MXA21.9*	AT5G38220.1	Alpha/beta-Hydrolases superfamily protein
Isoform0013869	*At1g52430*	AT1G52430.1	F6D8.35 protein
Isoform0063160	*At4g17480*	AT4G17480.2	Alpha/beta-Hydrolases superfamily protein
Isoform0062569	*At2g32520*	AT2G32520.4	Alpha/beta-Hydrolases superfamily protein
Isoform0052797	*SAMDC4*	AT5G18930.1	S-adenosylmethionine decarboxylase proenzyme 4
Isoform0014448	*AK1*	AT5G13280.1	Aspartokinase 1, chloroplastic
Isoform0062351	*CYSC1*	AT3G61440.1	Bifunctional L-3-cyanoalanine synthase/cysteine synthase C1, mitochondrial
Isoform0030597	*SAT2*	AT2G17640.1	Serine acetyltransferase 2
Isoform0062985	*FPGS2*	AT3G10160.1	Folylpolyglutamate synthase
Isoform0038474	*At2g40570*	AT2G40570.1	Submitted name: At2g40570

Isoform0053614	*CYCLASE2*	AT4G35220.1	Cyclase-like protein 2
Isoform0035742	*At3g20270*	AT3G20270.2	Putative BPI/LBP family protein At3g20270
Isoform0061596	*At3g02620*	AT3G02620.3	Stearoyl-[acyl-carrier-protein] 9-desaturase
Isoform0061683	*At3g29075*	AT3G29075.1	Submitted name: Glycine-rich protein
Isoform0036319	*ACC1*	AT1G36160.1	Acetyl-CoA carboxylase 1
Isoform0062607	*At2g17340*	AT2G17340.1	Damage-control phosphatase At2g17340
Isoform0012059	*FAR7*	AT5G22420.2	Fatty acyl-CoA reductase
Isoform0029287	*PLIP1*	AT3G61680.1	Phospholipase A1 PLIP1, chloroplastic
**Others metabolism (5)**			
Isoform0062871	*SCPL9*	AT2G23010.1	Serine carboxypeptidase-like 9
Isoform0029132	*MED33B*	AT2G48110.1	Mediator of RNA polymerase II transcription subunit 33B
Isoform0019259	*5MAT*	AT3G29590.1	Malonyl-CoA:anthocyanidin 5-O-glucoside-6''-O-malonyltransferase
Isoform0062246	*At5g28840*	AT5G28840.2	GDP-mannose 3,5-epimerase
Isoform0043965	*MJG14.10*	AT5G37170.1	O-methyltransferase family protein

Table S6. Ten lncRNAs involved in protein kinase and others signaling.

LncRNAs ID	Co-expressed mRNAs	mRNA ID	Proteins Encoded by Co-expressed mRNAs
Isoform0038381	*HIR2*	AT1G69840.6	Hypersensitive-induced response protein 2
Isoform0023648	*At5g12000*	AT5G12000.1	Protein kinase domain-containing protein
Isoform0063233	*At1g13050*	AT1G13050.2	Proline-rich receptor-like kinase
Isoform0005251	*PI4KB1*	AT5G64070.1	Phosphatidylinositol 4-kinase beta 1
Isoform0062015	*RLP12*	AT1G71400.1	Receptor-like protein 12
Isoform0062945	*At1g52600*	AT1G52600.1	Signal peptidase I
Isoform0062312	*SRP-54C*	AT1G48900.1	Signal recognition particle 54 kDa protein 3
Isoform0062313	*At2g45180*	AT2G45180.1	At2g45180
Isoform0063004	*AGB1*	AT4G34460.3	GTP binding protein beta 1
Isoform0015765	*SNF4*	AT1G09020.1	Sucrose nonfermenting 4-like protein
